# Dune soil nitrogen leaching for Chinese-yam cultivation: Impact of microbe-decomposable slow-release fertilizer

**DOI:** 10.1016/j.heliyon.2024.e30545

**Published:** 2024-05-03

**Authors:** Akira Endo

**Affiliations:** Faculty of Agriculture and Life Science, Hirosaki University, 3 Bunkyo-Cho, Hirosaki, Aomori, 036-8561, Japan

**Keywords:** Chinese-yam cultivation, Nitrogen leaching, Fast-acting and slow-release fertilizer, Excessive irrigation, Groundwater environment, Numerical analysis

## Abstract

Chinese yam production is thriving in Aomori Prefecture, a cold and snowy region in Japan. Recently, there has been an increasing risk of nitrogen leaching in Chinese-yam fields, which consist of sandy soil, due to localized torrential rain. The relationships between the type of fertilizer used for Chinese-yam cultivation, the amount of nitrogen (N) leaching, and the timing of leaching remain unknown. Therefore, this study aimed to fill this knowledge gap by investigating the effects of different fertilizers (fast-acting and/or slow-release fertilizer) and irrigation practices (conventional and/or excessive irrigation) in order to mitigate the detrimental impact of nitrogen leaching on groundwater quality. An enhanced mathematical model and the spatiotemporal dynamics of inorganic nitrogen concentration in soil pore water were evaluated the negative impact of nitrogen leaching on the groundwater environment was evaluated. The results showed that the combined use of slow-release fertilizers could significantly reduce nitrate-nitrogen concentration in soil-water, especially during the harvest season. This study demonstrated that cultivating Chinese yam with a fertilizer application system that includes the use of slow-release fertilizer can diminish the negative impact of nitrogen leaching on the groundwater environment, contributing to our understanding of sustainable agricultural practices in regions facing similar environmental challenges. Therefore, our findings represent an important advancement providing new approaches to maintaining productivity while mitigating the adverse impacts on groundwater environments, as well as offering guidelines for agricultural practices in regions facing similar environmental challenges.

## 1 Introduction

The Chinese-yam (*Dioscorea japonica* Thunb.), belonging to the family Dioscoreaceae, is classified into long, flat, and massive species according to its shape. *Dioscorea japonica* originates in China and is widely cultivated in Japan, South Korea, Taiwan, Vietnam, and other East Asian monsoon regions. Coursey (1981) reported that yams are not only a staple food consumed by approximately 155 million people worldwide but are also cultivated as cash crops and medicinal plants with high cultural value [1]. Chinese-yam production is particularly thriving in the Aomori Prefecture in Japan, which is a cold and snowy region that yields approximately 57,300 tons of Chinese yam in a planted area of 2,220 ha [2], accounting for the second-highest yield and largest planted area in Japan (MAFF, 2023). The long species type of Chinese yam is primarily cultivated in a large-scale upland fields (dune fields) in the northwestern Aomori Prefecture region, in a cold and snowy region reclaimed by the National Byobuzan Reclamation Construction Project. Nonetheless, the intensifying localized torrential rainfall has exacerbated the issues of nitrogen leaching and low-nitrogen available efficiency in Chinese yam cultivation.

Despite previous research falling short in addressing this matter, especially concerning the effects of different fertilizer types and irrigation conditions on nitrogen leaching, recent studies have shed light on the interplay between soil properties, fertilization methods, and the outcomes of yam cultivation. Cornet et al. (2022) reported that nitrogen leaching reduces yam growth by limiting soil nitrogen availability near the tuber in the initial growth stage [3]. Ma et al. (2019) investigated the impacts of two soil types (sandy and loessial soil) on the continuous cropping of Chinese yam in terms of the polysaccharide contents and reported that Chinese yam cultivated in sandy soil may have a higher glucose levels [4]. Akanji et al. (2019) proposed a statistical model for estimating the yield of yams (*Dioscorea alata*) using apparent soil electrical conductivity (EC) as an index and reported that the growth and yield of yams were affected by the interaction between soil physical and chemical properties [5]. Endo et al. (2018a) established a Chinese yam cultivation test plot with two types of irrigation conditions: (i) a conventional irrigation plot (control plot) and (ii) an excessive irrigation plot in the Byobuzan dune field in Aomori Prefecture, where the soil pore water quality at arbitrary depths was monitored during the cultivation period [6]. The authors showed that the NO_3_^−^ content was 100 mg L^−^^1^ or more in the control plot at a depth of 10–30 cm from mid-August to late August due to the accumulation and leaching and the accumulation of substances owing to precipitation and irrigation patterns. Conversely, in the excessive irrigation plot, the NO_3_^−^ content was 100 mg L^−^^1^ or more at an approximate depth of 50 cm from the end of August to the beginning of September. The authors also indicated that when localized torrential rains are expected, it is necessary to utilize slow-release fertilizers to reduce the amount of N leaching. In addition, under general cultivation conditions, the accumulation of a high NO_3_^−^ content of 40–80 mg L^−^^1^ was observed at a depth of 50 cm from late September to early November.

Therefore, the authors predicted that the snow cover from December to February of the following year might result in a remarkably NO_3_–N leaching together with infiltrated water as soon as snowmelt began in early spring. Jinno (2000) used lysimeters to analyze the nitrogen balance under 11 fertilization conditions, including top dressing in an in-dune Chinese-yam field [7]. Reducing the amount of infiltrated water highly effectively reduced the amount of N-leaching as the N-leaching amount was small in the year when the annual rainfall was low. These results indicate that the nitrate–nitrogen concentration in the groundwater below Chinese-yam fields in the dunes is likely to exceed the environmental standard of 10 mg L^−^^1^. However, under localized torrential rain, which recently increased, the interplay between the type of fertilizer used when cultivating Chinese yam, the amount of N leaching, and the timing of leaching has not been elucidated. However, under localized torrential rain, which recently increased, the interplay between the type of fertilizer used when cultivating Chinese yam, the amount of N leaching, and the timing of leaching has not been elucidated.

Therefore, the author elucidated the impact of fertilizer selection and irrigation methods on nitrogen leaching in Chinese-yam cultivation to achieve sustainable agricultural practices. Specifically, the aims of this study were to (i) apply the nitrogen transport model developed by Endo et al. (2018b) to the Chinese-yam field, and (ii) evaluate the negative impacts of nitrogen leaching on the groundwater environment using the slow-release fertilizer according to the spatiotemporal dynamics of inorganic nitrogen concentration in soil pore water, as calculated using the developed model [8]. Our findings may provide novel approaches to maintain productivity while mitigating adverse effects on groundwater environments, as well as offering guidelines for agricultural practices in regions facing similar environmental challenges. It is worth noting that this study addresses a critical knowledge gap concerning the impact of fertilizer selection and irrigation methods on nitrogen leaching in Chinese-yam cultivation, which is crucial for achieving sustainable agricultural practices in regions prone to localized torrential rainfall and facing environmental challenges. Furthermore, we introduced a novel approach by utilizing a nitrogen transport model to evaluate the negative impacts of nitrogen leaching on groundwater environments, offering insights into mitigating adverse effects while maintaining crop productivity in Chinese-yam cultivation. Finally, our findings might provide valuable insights for farmers and policymakers aiming for sustainable agricultural practices that maintain crop productivity while minimizing environmental impacts.

## 2 Materials and methods

### 2.1 Outline of the study field and measurement of the basic soil properties

**Fig. 1:**
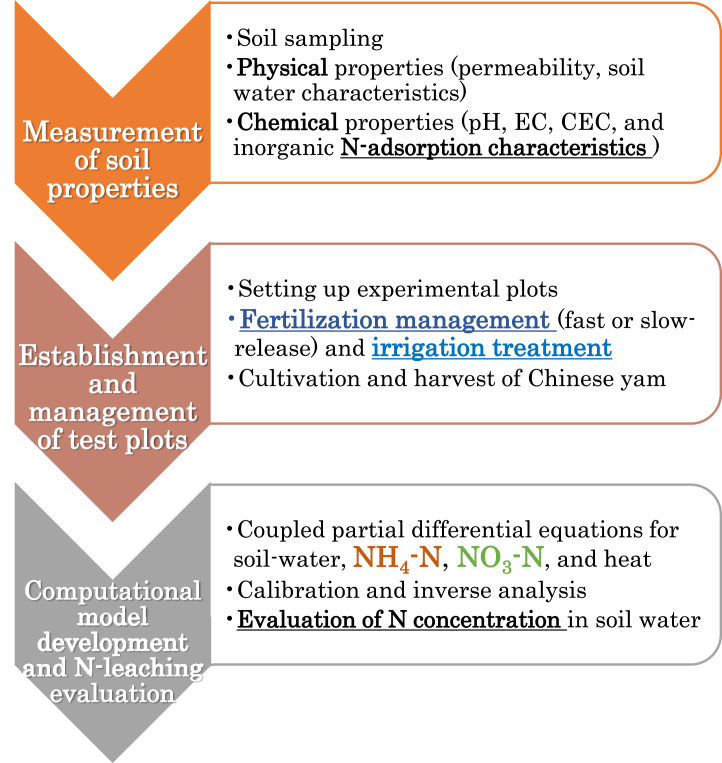
The research methodology flowchart.

**Fig. 2:**
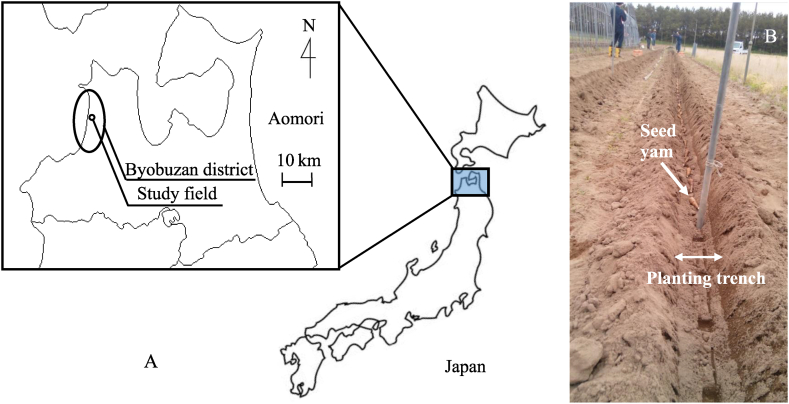
Study area of the Chinese yam cultivation field (A) and planting of seed yams in the studied cultivation field (B).

The research methodology flowchart is illustrated in Fig. 1. The study field was a large-scale upland field reclaimed in the dune area of the Byobuzan district (Fig. 2A) in Aomori Prefecture, Japan, at N40°56′50″, E140°19′43″ and an elevation of 26 m above sea level. The reference soil group was categorized as Arenosols [AR] according to the World Reference Base for Soil Resources (IUSS Working Group WRB, 2022) [9]. This area is located in the Tohoku region of Japan, which has a subarctic zone-humid climate and experiences approximately 300 cm of substantial snow accumulation in the winter season (from December to March of the following year). The average snow-melting period spans from late February to mid-March. One field lot (400 m × 150 m) encompassed 6 ha with sprinklers deployed at 15 m intervals. In addition, deep underground drainage (buried depth 150 cm, drainage culvert diameter 10–15 cm) was constructed at intervals of 30 m in the field.

In April 2015, the author excavated the soil surface to a depth of 150 cm for soil sampling. To determine the physical and chemical properties of the Chinese-yam field soil, undisturbed (100-mL soil core sample) and disturbed soil samples were collected at depths of 10, 30, 50, 70, 100, and 150 cm. Subsequently, soil particle density and size distribution were measured to determine the soil texture according to Japan Industrial Standards (JIS) A1204 and A1202, respectively. The saturated hydraulic conductivity and soil-water retention curves (SWRC) necessary to estimate unsaturated hydraulic conductivity were determined based on JIS A1218 and the Japanese Geotechnical Society (JGS) 0151, respectively. The parameters of the SWRC were calculated according to the van Genuchten (1980) model [10] as shown in Eq. [1] using non-linear curve fitting (Mathcad 15, PTC Inc.),

[1] $$\theta \left(\psi \right)=\theta_{r}+\frac{\theta_{s}-\theta_{r}}{{\left[1+{\left(\alpha \left|\psi \right|\right)}^{n}\right]}^{m}},\;m=1-\frac{1}{n}$$

where *θ* is volumetric water content (cm^3^ cm^−^^3^), *θ*_s_ is saturated volumetric water content (cm^3^ cm^−^^3^), *θ*_r_ is residual volumetric water content (cm^3^ cm^−^^3^), *ψ* is matric potential (-cmH_2_O),*α* is scaling parameter (cm^−^^1^), and *n* is shape parameter.

These tests for soil physical properties were conducted according to the Japanese Geotechnical Society (2010) [11]. The pH and EC of soil subjected to extraction with 1:2.5 and/or 1:5 deionized water were measured using the glass electrode (B-712, Horiba) and electrical conductivity methods (B-771, Horiba), respectively. The cation exchange capacity (CEC) and phosphate absorption capacity were measured using the semi-micro Kjeldahl (SuperKjel-1300, Actac) and phosphovanadomolybdate methods, respectively. To calculate the C/N ratio, total carbon (T-C) and total nitrogen (T-N) were quantified using an elemental analyzer (Vario EL cube, Elementar, Germany). For numerical model calculation, parameters such as soil physical and chemical properties, N-uptake characteristics during the Chinese-yam growing period, and mineralization characteristics of the delayed-release fertilizer (H-CDU) were input into the model.

### 2.2 Installation of test plots, cultivation management, and irrigation treatment

In May 2016, in the Chinese-yam cultivation test plot, a planting trench for growing the yam downward was formed to a depth of approximately 1 m using a double-type trencher. Subsequently, two types of test plots, conventional (control) and excessive irrigation, were installed. After that, soil-water sampling tubes were installed at depths of 10, 30, 50, 70, 100, and 150 cm, and soil-water samples were collected by suction with a syringe at a frequency of approximately once every 2 weeks. Then, NH_4_–N and NO_3_–N concentrations in sampled soil-water were quantified using ion chromatography (Endo et al., 2018c). Further, in June 2016, seed yams (breed variety: Ensikei-6) with a length of approximately 10 cm were transplanted to each test plot (Fig. 2B).

Finally, in November, the Chinese yams were harvested using a conveyor trencher. The fertilizers listed in Table 1 were surface-fertilized on the flat ridges during the germination, early growing, middle growing, and thickening stages. The fertilizers named “exclusively for Chinese yam” and “mixed fertilizer” are fast-acting granular compound fertilizers. Hyper CDU (H-CDU), a medium-term element-type fertilizer of acetaldehyde condensation urea (2-oxo-4-methyl-6-ureido hexahydropyrimidine), has the characteristic that mineralization continues for 60–90 days after fertilization.

**Table 1:** Fertilization date, fertilizer constituents, and fertilization amount for Case-1.

Growing stage Fertilization date	Fertilizer constituents N–P–K (%)	Fertilization amount (kgN ha^−^^1^)
Germination stage Jun 29, 2016	Exclusively for Chinese yam 14-17-10	143
Early growing stage July 13, 2016	Mixed fertilizer 10-12-7	120
Middle growing stage July 26, 2016	H-CDU 15-8-8	60
Thickening growing stage August 8, 2016	H-CDU 15-8-8	60

The mineralization mechanism of CDU [12] is shown in Eq. [2].

**Figure:**
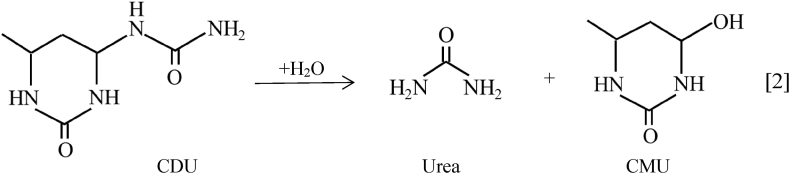

In the numerical calculation, Case-1 and Case-2 fertilizers, which are listed in Table 1, Table 2, respectively, were applied based on their fertilization conditions. Case-1 (Table 1) fertilizer was applied during the field growing experiment (Endo et al., 2018) [8]. The Case-2 fertilizer listed in Table 2 replaced the fertilizers of Case-1 with H-CDU.

**Table 2:** Fertilization date, fertilizer constituents, and fertilization amount for Case-2.

Growing stage Fertilization date	Fertilizer constituents N–P–K (%)	Fertilization amount (kgN ha^−^^1^)
Germination stage Jun 29, 2016	H-CDU 15-8-8	143
Early growing stage July 13, 2016	H-CDU 15-8-8	120
Middle growing stage July 26, 2016	H-CDU 15-8-8	60
Thickening growing stage August 8, 2016	H-CDU 15-8-8	60

In general, rainwater is the main water source for Chinese-yam cultivation. However, in the case of sandy soil with low water retention characteristics, sprinkler irrigation is required in dry soil conditions as the crop water supply is insufficient with rainwater alone. In the field irrigation plan of the Byobuzan area, the amount of irrigation per time was set to 16 mm (MAFF Tohoku Regional Agricultural Administration Office, 1991) [13]. In both test plots, sprinklers were activated five times from May to July, and a total of 80 mm (16 mm × 5 times) of irrigation was applied. In contrast, in the excessive irrigation plot, in addition to sprinkler irrigation, excessive irrigation (210 mm/event) by shower sprinkling was conducted five times from August to September to irrigate a total of 1,050 mm (210 mm × 5 times). This excessive irrigation mimics the frequent occurrence of localized heavy rainfall due to the accelerated progress of climate change observed in recent years. The author set up excessive irrigation to consider a daily rainfall of 200 mm d^−1^, which is expected to occur once every 100 years (Japan Meteorological Agency, 2022) [14].

### 2.3 Parameters of the adsorption characteristics of inorganic-N for cultivated soil

#### 2.3.1 Measurement method of adsorption isotherm

In the adsorption isotherm experiment, soil samples collected in the Chinese-yam cultivation field from depths of 10–150 cm in April 2016 were used. The batch adsorption test proposed by Tani et al. (2004) [15] was applied, and the relationship between the equilibrium concentration and the adsorption amount of inorganic nitrogen was quantified according to the procedure described by Endo et al. (2009) [16]. First, approximately 2 g of the dried soil sample was placed in a 50 mL centrifuge tube with a lid. Next, to saturate the adsorption site on the particle surface of the soil sample with Na^+^ and Cl^−^, approximately 30 mL of 1 mol L^−1^ sodium chloride solution was placed in a centrifuge tube, shaken for 1 h, and the supernatant was separated via centrifugation and excluded. Approximately 30 mL of deionized water was placed in the centrifuge tube, which was stirred and shaken well prior to centrifugation, and the supernatant was excluded. Next, 5 mL of an aqueous solution of ammonium nitrate (NH_4_NO_3_) with initial concentrations of 1, 5, 10, 25, and 50 mmol L^−1^ was placed in a centrifuge tube containing a washed sample, and then a small amount of sodium hydroxide (NaOH) solution was added to the centrifuge tube and adjusted to pH 6.00. Next, in a room with a constant temperature of 25 °C, a centrifuge tube with a lid containing a pH-adjusted sample was shaken for 2 h. Next, the pH was measured immediately to confirm that the pH of the soil solution did not change significantly when shaking was completed. The state of adsorption at the completed shaking was defined as the equilibrium adsorption. Next, the supernatant extracted via centrifugation was filtered using a syringe filter with a pore size of 0.45 μm. Finally, the filtrate was diluted with ultrapure water, and NH_4_^+^ and NO_3_^−^ levels were quantified using ion chromatography (ICS-90, Dionex).

#### 2.3.2 Analysis method of adsorption isotherm characteristics

The Redlich–Peterson type and Freundlich adsorption isotherms were applied to the adsorption model that expresses the relationship between the adsorption amount and the equilibrium concentration of inorganic nitrogen obtained by the adsorption experiment. The Freundlich (1906) type adsorption isotherm [17] shown in Eq. [3] has a good empirical match between the adsorption amount and the equilibrium concentration (Wu et al., 2002; Gutierrez and Fuentes, 1993) [18,19] and was applied to the adsorption of nitrate-nitrogen (NO_3_–N).

[3] $$s\left(c\right)={K_{F}c}^{1/n}$$

where *s* is the adsorption amount (mg g^−1^), *c* is the equilibrium concentration (mg cm^−3^), *K*_*F*_ is the relative adsorption capacity [mg^1−(1/*n*)^ (cm^3^)^1/*n*^ g^−1^], and *n* is the adsorption strength coefficient.

The Redlich–Peterson (1959) type adsorption isotherm [20] shown in Eq. [4] is known as a hybrid model in which the numerator of Eq. [4] is linearly dependent on the concentration, and the denominator is expressed by an exponential function of the equilibrium concentration (Chu et al., 2024; Wu et al., 2010) [21,22]. Eq. [4] was applied to the adsorption of ammonium nitrogen (NH_4_–N).

[4] $$s\left(c\right)=\frac{K_{R}c}{1+\alpha_{R}c^{\beta }}$$

where *s* is the adsorption amount (mg g^−1^), *c* is the equilibrium concentration (mg cm^−3^), *K*_R_ is the adsorption coefficient (cm^3^ g^−1^), *α*_R_ is the adsorption parameter (cm^3^ mg^−1^), and *K*_*R*_*/a*_*R*_ is the adsorption capacity. The dimensionless adsorption parameters *β* = 1 and *α*_R_ = 0 represent the Langmuir and Henry types, respectively. Therefore, this adsorption model incorporates the characteristics of both types of adsorption isotherms and is an empirical model expressed using three types of parameters to correct inaccuracies. The adsorption mechanism is unique, and although it does not follow the ideal single-layer adsorption represented by the Langmuir model (Langmuir, 1918) [23], the relationship between the adsorption amount and the NH_4_–N equilibrium concentration of soil can be expressed relatively well. When calculating the curve parameters of the adsorption model equations, these adsorption data were fitted to the adsorption models described in Eqs. [3] and [4] using non-linear curve fitting (Mathcad 15, PTC Inc.).

### 2.4 Computational model description

#### 2.4.1 Governing equations

**Fig. 3:**
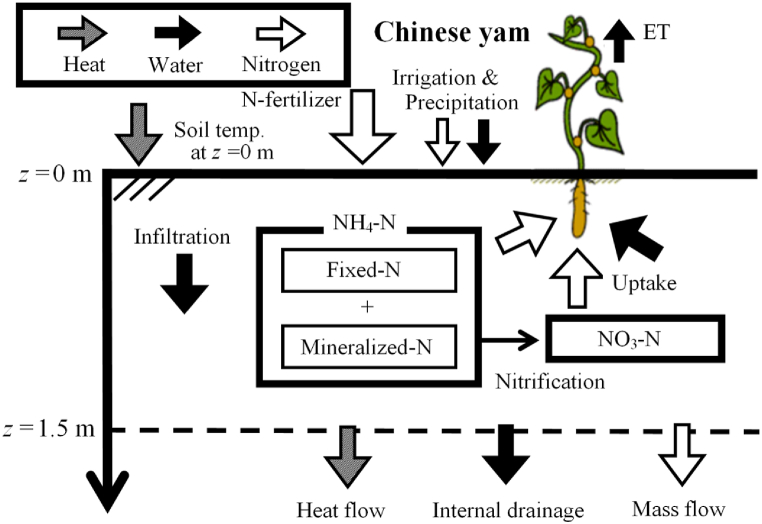
Schematic view of the model for calculating inorganic-N (NH_4_–N and NO_3_–N) in the Chinese yam cultivation field. ET, evapotranspiration.

Mass (e.g., soil-water and inorganic nitrogen) and energy (e.g., heat) transportation can be described in terms of a partial differential equation (PDE) according to the method described by Endo et al. (2009) [16]. The original numerical model is a nitrogen transport model designed for an apple orchard. However, this study focuses on nitrogen migration in Chinese-yam cultivation fields, and the root system distribution and the timing of nitrogen uptake differ between apples and Chinese yams. For this reason, the nitrogen transport model for apple orchards developed by Endo et al. (2018) [8] was modified for application to the Chinese yam cultivation field. An attempted to rationalize the numerical model was conducted by applying the root system distribution of apple trees, such as perennial orchard trees, to that of the Chinese yam, a non-permanent crop, according to the growth process. A schematic of the computational model is shown in Fig. 3.

The author used FlexPDE V7 (PDE Solutions Inc.) to adapt the computational model and solve the PDEs for the yam-cultivated field. The mass and energy components are divided into the transport of (i) soil water, (ii) inorganic nitrogen (NH_4_–N and/or NO_3_–N), and (iii) heat. FlexPDE software has previously been applied to conduct mass and energy transportation analyses in soils (Endo et al., 2009; Endo et al., 2013; Endo et al., 2017; Endo et al., 2018b) [8,16,24,25]. The governing equations are expressed using the Richards equation (Eq. [5]), the convection–dispersion equation for inorganic-N (Eqs. [9] and [12]), and the heat conduction equation (Eq [15]). The governing equations of soil-water transport are expressed as the Richards equation:

[5] $$c_{w}\frac{\partial \psi }{\partial t}=\frac{\partial }{\partial z}\left(K_{u}\left[\frac{\partial \psi }{\partial z}-1\right]\right)-{ET}_{a}S\left(z,t\right)$$

where *ψ* is the matric potential (cm H_2_O), *t* is the elapsed days (d), *c*_w_ is the soil-water capacity (cm^−1^), *z* is the soil depth (cm), *K*_*u*_ is the unsaturated hydraulic conductivity (cm s^−1^), *ET*_a_ is the actual evapotranspiration (cm d^−1^), and *S* (*z, t*) (Eq [6]) is the root density distribution for Chinese yam (cm^−1^) proposed by Endo et al. (2018c) [26],

[6] $$S\left(z,t\right)=\frac{a\left(t\right)}{b\left(t\right)+c\left(t\right)\;\exp\left[d\left(t\right)\;z\right]\;}$$

where *a*(*t*), *b*(*t*), *c*(*t*), and *d*(*t*) are parameters that determine the shape of the root system distribution.

The actual evapotranspiration *ET*_a_ is represented as Eq. [7], which expresses the evapotranspiration as a function of the volumetric water content *θ* (cm^3^ cm^−3^) and *t*:

[7] $${ET}_{a}=\left\{ \begin{array}{cc} \;f\left(\theta \right){K_{c}ET}_{0}\;\sin\left(2\pi t-\frac{\pi }{2}\right) & \left(\frac{\pi }{2}<t<\frac{3\pi }{2}\right)\\ 0 & \text{otherwise} \end{array}\right.$$

[8] $$f\left(\theta \right)=\left\{ \begin{array}{cc} \;1 & \left(\theta >\theta \left({-10}^{2.0}\text{cm}H_{2}O\right)\right)\\ \;\frac{\theta -\theta \left({-10}^{2.7}\text{cm}H_{2}O\right)}{\theta \left({-10}^{2.0}\text{cm}H_{2}O\right)-\theta \left({-10}^{2.7}\text{cm}H_{2}O\right)} & \left(\theta \left({-10}^{2.7}\text{cm}H_{2}O\right)\leq \theta \leq \theta \left({-10}^{2.0}\text{cm}H_{2}O\right)\right)\\ \;0 & \left(\theta \leq \theta \left({-10}^{2.7}\text{cm}H_{2}O\right)\right) \end{array}\right.$$

where *K*_c_ is the crop coefficient (0.7–1.0) for Chinese yam according to MAFF (1997), *ET*_0_ is the reference evapotranspiration (cm d^−1^) (Kuwagata et al., 2009) [27], and *f*(*θ*) is expressed as a function of the volumetric water content at the soil surface. The numbers in parentheses are the matric potentials. Note that Eq. [8] assumes a falling stage of evaporation (Endo et al., 2009) [16].

For the transportation of NH_4_–N and NO_3_–N, the advection-dispersion equation described in Eqs. [9] and [12] was adopted. These PDEs consider the phenomena of N-formation change, such as mineralization and nitrification, as well as N uptake by plant roots. A description of the NH_4_–N transportation equation is given in Eq. [9]:

[9] $$\frac{\partial \left(c_{1}\theta +\rho_{d}s_{1}\right)}{\partial t}=\frac{\partial }{\partial z}\left(\theta D_{1}\frac{\partial c_{1}}{\partial z}\right)-\frac{\partial \left(J_{w}c_{1}\right)}{\partial z}-k_{\text{nit}}\left(c_{1}\theta +\rho_{d}s_{1}\right)-u_{{\text{NH}}_{4}‐N}S\left(z,t\right)\Delta U+N_{\text{mine}}$$

where *c*_1_ is the NH_4_–N concentration of pore water; *s*_1_ is the adsorption amount of NH_4_–N; $u_{{\text{NH}}_{4}‐N}$ is the cumulative uptake of NH_4_–N by yam roots (mg cm^−2^); Δ*U* is the temporal distribution concerning the N-uptake, as shown in Eq. [14] (d^−1^); *N*_mine_ is the mineralized N amount (mg cm^−3^ d^−1^) at depths of 0–10 cm; *k*_nit_ is the nitrification rate coefficient expressed as functions of elapsed time and soil temperature [16,28], as shown in Fig. S1 and Eq. [10] (d^−1^) and Eq. [11]; *ρ*_d_ is the bulk density of the soil (g cm^−3^); *D*_1_ is the hydrodynamic dispersion coefficient of NH_4_–N (cm^2^ d^−1^); *J*_w_ is the water flux density (cm^3^ cm^−2^ d^−1^).

[10] $$k_{nit}\left(t,T\right)=\gamma \left(T\right)e^{-0.224t}$$

[11] $$\gamma \left(T\right)={1.189\times 10}^{-3}e^{0.152T}$$

The author periodically surveyed the soil surface of the Chinese yam upland field, and no undergrowth of legume plants was observed; therefore, nitrogen fixation was neglected. In addition, the set value of the annual N-mineralized amount (*N*_mine_) was 0.142 mg N cm^−2^ using the method of days transformed to standard temperature, as proposed by Sugihara et al. (1986) [29] and the reaction kinetic parameters of Furue and Uwasawa (2001) [30]. The description of the NO_3_–N transportation equation is given in Eq. [12]:

[12] $$\frac{\partial \left(c_{2}\theta +\rho_{d}s_{2}\right)}{\partial t}=\frac{\partial }{\partial z}\left(\theta D_{2}\frac{\partial c_{2}}{\partial z}\right)-\frac{\partial \left(J_{w}c_{2}\right)}{\partial z}+k_{\text{nit}}\left(c_{1}\theta +{\;\rho }_{d}s_{1}\right)-u_{{\text{NO}}_{3}‐N}S\left(z,t\right)\Delta U$$

where *c*_2_ is the NO_3_–N concentration of pore water, *s*_2_ is the adsorption amount of NH_4_–N, $u_{{\text{NO}}_{3}‐N}$ is the cumulative uptake of NO_3_–N by yam roots (mg cm^−2^), and *D*_2_ is the hydrodynamic dispersion coefficient of NO_3_–N (cm^2^ d^−1^).

The nitrogen uptake expressed as the fourth term on the right-hand side of Eqs. [9] and [12] can be expressed as the product of the relative N uptake per unit time (the time derivative of the red-colored curve in Fig. S2 and the total N uptake (1.215 mg cm^−2^). Therefore, the area enclosed by the red-colored curve and the time axis represents the total N-uptake.

In this study, the tuber length and mass of the yam were measured at arbitrary dates during the growth period of the yam; however, data on the daily change in nitrogen uptake was not obtained. For this reason, we assumed the temporal changes in tuber length and mass of the yam to be due to cumulative N-uptake by the yam (relative values expressed as 0 < *U* < 1); then, non-linear curve fitting was performed using Eq. [13] for these data points.

The temporal change in the assumed relative cumulative N uptake (*U*) and its time derivative (Δ*U*) are expressed as follows:

[13] $$U\left(t\right)=\frac{1}{1+e^{\left[-K_{1}\left(t-m_{1}\right)\right]}}$$

[14] $$\Delta U\left(t\right)=\frac{dU\left(t\right)}{dt}=\frac{K_{1}e^{K_{1}\left(m_{1}-t\right)}}{{\left(1+e^{K_{1}\left(m_{1}-t\right)}\right)}^{2}}$$

where the fitting parameters *K*_1_ and *m*_1_ are 0.08 and 75.97, respectively.

The description of the conductive and advective heat transfer equation (Luce et al., 2013) [31] is given in Eq. [15].

[15] $$\rho c\frac{\partial T}{\partial t}=\frac{\partial }{\partial z}\left(\lambda \frac{\partial T}{\partial z}\right)-J_{w}{\left(\rho c\right)}_{w}\frac{\partial T}{\partial z}$$

where *Τ* is the soil temperature (°C), *ρc* is the soil volumetric heat capacity (MJ m^−3^ K^−1^), (*ρc*)_w_ is the volumetric heat capacity of pore water (MJ m^−3^ K^−1^), and *λ* is the soil thermal conductivity (W m^−1^ K^−1^).

#### 2.4.2 Analytical domain, initial conditions (ICs), and boundary conditions (BCs)

To solve the partial differential equations expressed in Eqs. [5], [9], [12], and [15], the ICs and BCs are required. The IC profiles on June 1, 2016, of the volumetric water content, NH_4_–N and NO_3_–N concentrations, and soil temperature are shown in Fig. S3.

Details of ICs and BCs used for numerical analyses are provided in the Supplementary Material.

#### 2.4.3 Inverse analysis of the adsorption isotherm parameters for NH_4_–N

Generally, the adsorption characteristics of nutrient salts in sandy soil are low. Nakamura et al. (2004) [32] conducted a batch adsorption test using a (NH_4_)_2_SO_4_ solution and reported that the adsorption amount of NH_4_–N was approximately 0.04 mg NH_4_–N g^−1^ at an equilibrium concentration of 0.5 mg NH_4_–N cm^−3^. Although the soil of the yam cultivation field is sandy, the adsorption amount of the equilibrium condition is approximately ten times greater than that reported by Nakamura et al. (2004) [32]. In actual field conditions, after fertilization and after the occurrence of precipitation or irrigation, N leaching may also occur before the equilibrium condition is reached. Among the various parameters applied to the numerical calculations, the adsorption isotherm of NH_4_–N described in Eq. [3] had a significant impact on the inorganic nitrogen concentration. Therefore, considering the sensitivity of the calculation results to this adsorption isotherm, an inverse analysis of the adsorption isotherm parameters was performed. Specifically, focusing on the adsorption isotherm parameters for NH_4_–N described in Eq. [3], the calibrated adsorption parameters *K*_R_′, *α*_R_′, and *β*′ listed in Table 3 for input in the numerical model were obtained by trial and error for the adsorption parameters in Eq. [3], so that the calculated concentrations approximated the spatiotemporal distributions of the measured concentrations.

**Table 3:** Adsorption isotherm parameters of NH_4_–N and NO_3_–N.

Depth *z* (cm)	*K*_f_	*n*	*K*_R_	*α*_R_	*β*	*K*_R_′	*α*_R_′	*β* ′
10	0.195	0.599	113.24	233.67	0.846	25.59	263.31	0.842
30	0.374	0.510	110.91	229.97	0.794	25.80	266.97	0.791
50	0.343	0.599	38.68	92.34	0.904	21.87	252.92	0.849
70	0.285	0.657	122.68	232.25	0.805	29.47	278.16	0.801
100	0.246	0.785	117.29	230.44	0.792	27.85	273.04	0.788
150	0.369	0.672	104.95	237.28	0.795	24.57	277.36	0.792

*K*_R_′, *α*_R_′, and *β* ′ are the calibrated parameters for NH_4_–N.

## 3 Results

### 3.1 Physical and chemical properties of soils

Fig. S5 shows the soil physical properties of the soil of the Chinese-yam field and the vertical distribution of van Genuchten's soil-water characteristic curve parameters calculated using the non-linear curve fitting method.

According to the IUSS method, the soil texture taxonomy was Sand (S), and the dry density range was 1.34–1.56 g cm^−^^3^. The soil-water content was 7–14 %, the saturated hydraulic conductivity was high in the range of 1.68 × 10^−3^–5.68 × 10^−3^ cm s^−^^1^, and the permeability tended to increase as the depth increased.

Fig. S6 shows the soil-water characteristic curve at each depth. The data points and curves represent the measured and fitted curves using the van Genuchten (1980) [10] model, respectively. As shown in Fig. S5, the saturated volumetric water content and the residual volumetric water content were approximately 0.33 and 0.01 cm^3^ cm^−^^3^, respectively, and the VG parameters α and n were approximately 0.02 and 2.66, respectively, with the characteristics of sandy soil confirmed.

Fig. S7 shows the vertical distribution of the soil chemical properties. The pH ranged from 6.11 to 6.45, which satisfied the target value range for improving the soil chemical properties of the Chinese-yam cultivation (pH 6.0–6.5). The EC ranged from 0.008 to 0.035 mS cm^−^^1^, which were exceptionally low EC levels. The average CEC was approximately 7.6 cmol kg^−^^1^, indicating the low nutrient retention capacity peculiar to sandy soil. The T-C and T-N represented low average values of 0.46 % and 0.04 %, respectively (average C/N = 13.2). Based on the above physical properties of the sandy soil, it might be necessary to artificially provide fertilizer components to the soil when cultivating Chinese-yam.

### 3.2 Adsorption isotherm characteristics of inorganic nitrogen

**Fig. 4:**
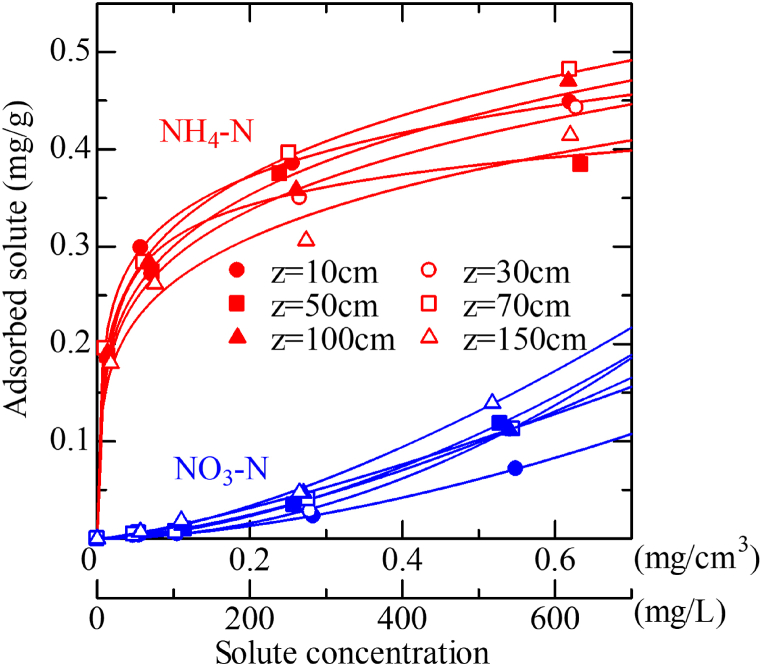
Adsorption isotherm of NH_4_–N and NO_3_–N for each depth. The red-colored and blue-colored lines represent NH_4_–N and NO_3_–N, respectively. (For interpretation of the references to color in this figure legend, the reader is referred to the Web version of this article.)

The adsorption isotherms of NH_4_–N and NO_3_–N at different depths and their fitted parameters based on Eqs. [3] and [4] are shown in Fig. 4 and Table 3, respectively. The data points and curves represent the measured and fitted curves, respectively. In the range of 0–600 mg L^−^^1^, the adsorption isotherm of NH_4_–N (represented by red color) and NO_3_–N (indicated by blue color) tended to have convex and concave shapes, respectively. Of note, these shapes did not depend on soil depth. The adsorption amount of ammonium nitrogen corresponding to the low concentration range of NH_4_–N (61–76 mg L^−^^1^) in the soil solution was 0.27–0.30 mg g^−^^1^. In contrast, the amount of NO_3_–N adsorbed in the low concentration range (47–67 mg L^−^^1^) was minimal (0.003–0.007 mg g^−^^1^). Based on this, the mobility of NO_3_–N at the solid-liquid interface corresponds to 43–90 times that of NH_4_–N using the ratio of the adsorbed amounts of both in the concentration range. Therefore, it can be inferred that even if there is rainfall or irrigation after surface fertilization, the movement of NH_4_–N is delayed, and it is easy to retain near the soil surface. The adsorption behavior of inorganic nitrogen in sandy soil collected in the Chinese-yam cultivation field showed a similar tendency to that in gray lowland soil and gravelly brown forest soil (Endo et al., 2017; Endo, 2021) [25,33].

### 3.3 Numerical calculation results of water and inorganic nitrogen transport in soil

#### 3.3.1 Behavior of soil moisture and inorganic-N

**Fig. 5:**
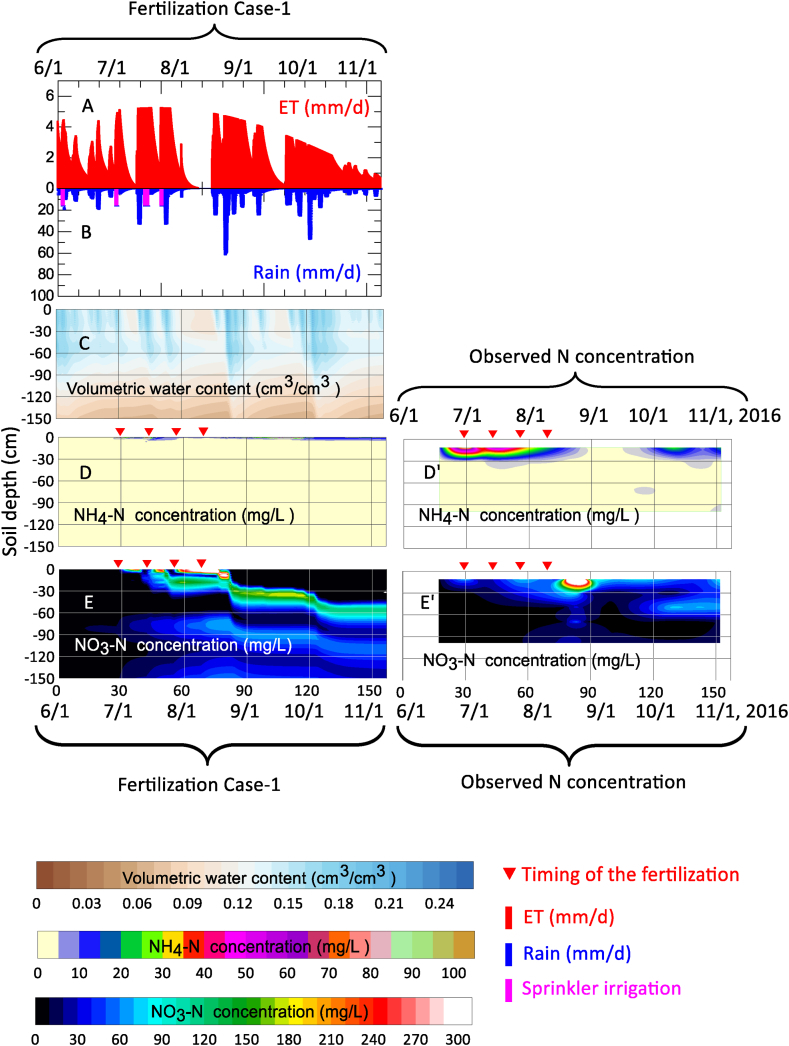
Evapotranspiration (A), precipitation and/or irrigation (B), numerical calculation results of the volumetric water content (C), NH_4_–N concentration (D), and NO_3_–N concentration (E) for Case-1 of fertilization conditions on the conventional irrigation plot. The measured NH_4_–N and NO_3_–N concentrations are shown in (D′) and (E′).

Fig. 5A–5E shows the evapotranspiration, amount of precipitation and/or irrigation, and numerical calculation results of the volumetric water content, NH_4_–N concentration, and NO_3_–N concentration, respectively, under Case-1 fertilization and the conventional irrigation conditions. In addition, Fig. 5D' and 5E' shows the measured NH_4_–N and NO_3_–N concentrations reported by Endo et al. (2018c) [26].

Focusing on the isopleths of the volumetric water content shown in Fig. 5C, wetting behavior during precipitation and/or irrigation and drying behavior during the period of no precipitation were observed. Regarding the wetting behavior, a wetting front with a volumetric water content of 0.17 cm^3^ cm^−^^3^ was formed to a depth of 76 cm by infiltrated water caused by the 70 mm of total rainfall that occurred during August 22–23, 2016 (the thickening growing stage).

In addition, intermittent rainfall after September 20 (t > 111 days) formed a wetting front of θ = 0.17 cm^3^ cm^−^^3^ to a depth of 74 cm on October 1 (t = 124 days). In contrast, for drying behavior, the volumetric water content of the soil layer z < 30 cm during the non-precipitation period from July 31 to August 14 decreased to θ = 0.08 cm^3^ cm^−^^3^. Focusing on z > 100 cm during the non-precipitation period, the dry domain, represented as θ < 0.08 cm^3^ cm^−^^3^ in Fig. 5C, tended to transition from a deep position to a shallow position over time due to internal drainage.

**Fig. 6:**
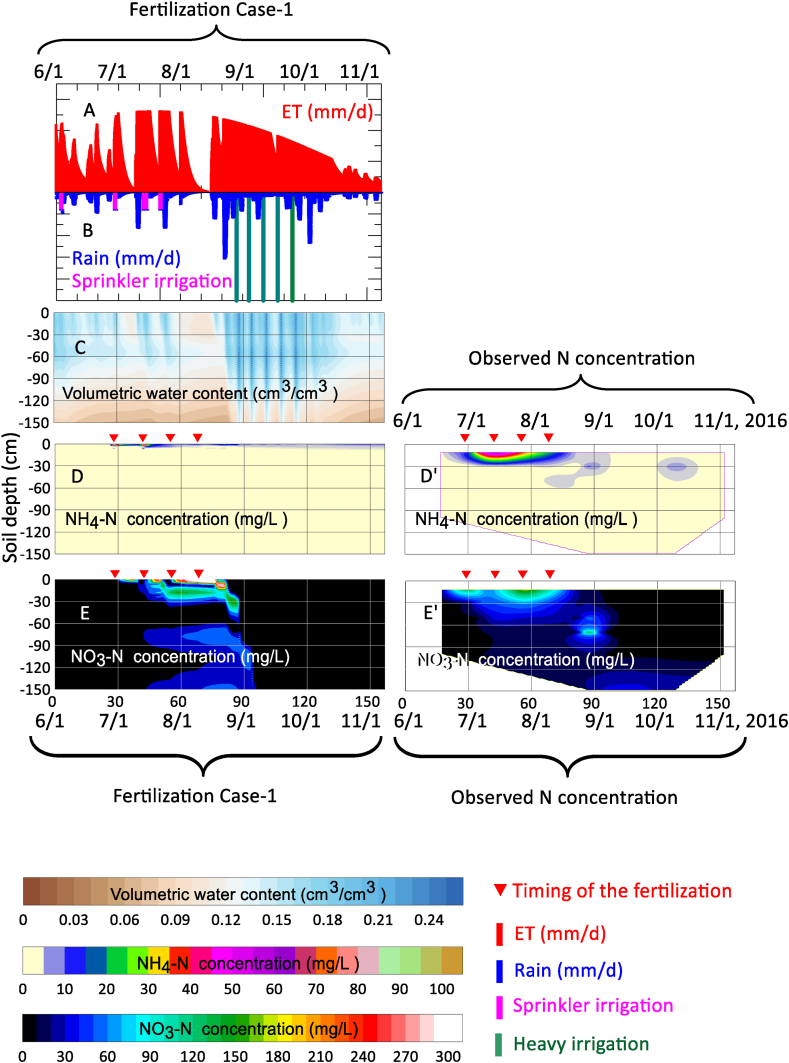
Evapotranspiration (A), precipitation and/or irrigation (B), numerical calculation results of the volumetric water content (C), NH_4_–N concentration (D), and NO_3_–N concentration (E) for Case-1 of fertilization conditions on the excessive irrigation plot. The measured NH_4_–N and NO_3_–N concentrations are shown in (D′) and (E′).

Fig. 5D and 5D′ show the isopleths of the NH_4_–N concentration in the soil pore water. In the Case-1 fertilizer application conditions shown in Fig. 5D, the NH_4_–N concentration on the soil surface rapidly increased to up to 20 mg L^−^^1^ at the time of application of the quick-acting fertilizer on June 29. Subsequently, the NH_4_–N concentration decreased with time due to nitrification. After applying H-CDU on July 26, no rapid increase in NH_4_–N concentration was observed, and mineralization continued until harvest day at approximately t < 153 days. In the measured concentration shown in Fig. 5D’, the concentration transitioned from 10 to 40 mg L^−^^1^ at 10 < z < 30 cm after fertilization. These results indicate that when the NH_4_–N adsorption isotherm parameters listed in Table 3 are applied to the numerical calculations, the calculated results are inconsistent with the measured NH_4_–N concentrations. Therefore, considering that the fertilized nitrogen is leached into NO_3_–N through the nitrification process, it is suggested that the adsorption conditions shown in Fig. 4 cannot accurately represent the inorganic nitrogen transport. Focusing on the behavior of the calculated NO_3_–N concentration illustrated in Fig. 5E, the NO_3_–N generated by nitrification after fertilization was gradually leached over time with step-like changes due to percolated water (the light sky blue-colored part in Fig. 5C after mid-August) caused by rainfall. However, for the measured NO_3_–N concentration illustrated in Fig. 5E’, NO_3_–N formed a concentration range of 200–300 mg L^−^^1^ at the depth from 10 to 20 cm in late August. Furthermore, there was a notable tendency of NO_3_–N accumulation observed at a depth of 50 cm following the month of October, which implies a potential risk of N leaching occurring abruptly as the snow melts in the subsequent spring. Fig. 6A–6E shows the evapotranspiration, amount of precipitation and/or irrigation, numerical calculation results of volumetric water content, NH_4_–N concentration, and NO_3_–N concentration, respectively, under Case-1 fertilization and the excessive irrigation conditions.

Focusing on the isopleths for volumetric water content shown in Fig. 6C, drying progressed rapidly after the wetting front of θ = 0.17 cm^3^ cm^−^^3^ reached a depth of 130 cm after a total of five heavy irrigations that corresponded to localized torrential rains (t = 86, 94, 101, 108, and 115 days). It was assumed that since the study field included sandy soil with high permeability and low water retention, the soil was dried because of the predominance of internal drainage after heavy irrigation. The calculation results in Fig. 6C show that five light-blue vertical lines were formed from the soil surface to a depth of 150 cm due to excessive irrigations (210 mm × 5 times) from the end of August. The maximum volumetric water content at a depth of 150 cm during the excessive irrigation period was about 0.15 cm^3^ cm^−^^3^ (corresponding to approximately −110 cm H_2_O for matric potential), which is approximately 50 % of the saturated volumetric water content value. Focusing on the behavior of NO_3_–N, the calculated results in Fig. 6E showed that the NO_3_–N accumulated at 30–40 cm was immediately leached to a depth of 100 cm by the first heavy irrigation in late August. However, the specific observation illustrated in Fig. 6E', indicates that NO_3_–N was leached out during the first heavy irrigation and subsequently accumulated at a depth of approximately 70 cm. This finding implies that the mathematical model formulated in this study failed to accurately replicate this significant advection phenomenon.

#### 3.3.2 Behavior of inorganic-N owing to model calibration

After the application of fast-acting fertilizer in Case-1, the NH_4_–N concentration at depths of 0–7 cm continuously increased until early November. Compared with the result before the model calibration (Fig. 5D), this result is closer to the behavior of the measured NH_4_–N (Fig. 5D').

In contrast, when only the delayed-release fertilizer was applied, as shown in Case-2, the NH_4_–N concentration and concentration distribution gradually increased with increasing depth, and mineralization continued until the yam harvesting time (approximately t = 157 days). As shown by the adsorption isotherm characteristics in Fig. 4, NH_4_–N was remarkably adsorbed on soil particles even at low concentrations; thus, no leaching behavior of NH_4_–N was observed.

Under the Case-1 fertilization conditions, the NO_3_–N concentration at depths of 0–30 cm increased beginning at 40 days after seed yam planting and reaching 200 mg L^−^^1^ at approximately 70 days, owing to the nitrification that occurred after fertilization. The downward transport of NO_3_–N reflected rainfall and irrigation. From mid- to late August, a high concentration of over 250 mg L^−^^1^ was reached in the 10 < z < 20 cm soil layer. Then, a total of 70 mm of supplied rainwater from August 22–23 caused the high-concentration front of NO_3_–N to move 20 cm downward, which resulted in a concentration of more than 150 mg L^−^^1^ in the 30 < z < 40 cm region during 88 < t < 122 days. Finally, at t = 157 days at the harvesting stage, high concentrations of NO_3_–N above 70 mg L^−^^1^ were formed in the soil layer region of 50 < z < 70 cm. The tendency of NO_3_–N to accumulate in the soil after yam harvesting was similar to the measured concentration distribution shown in Fig. 5E'.

Murphy et al. (2024) [34] conducted numerical simulations to investigate the leaching of inorganic nitrogen in sandy soil using the Hydrus model. In order to capture the complexity of these processes, they employed zero-order reaction models, first-order reaction models, and conditional kinetic models for biogeochemical reactions. The authors reported a strong agreement between the measured and calculated concentrations of nitrate–nitrogen in soil–water when applying the first-order reaction model utilized in this study. Given that this study also examined soils with similar sand characteristics and applied a first-order reaction to biogeochemical reactions, the reliability of the numerical calculations is, therefore, reasonably sound.

**Fig. 7:**
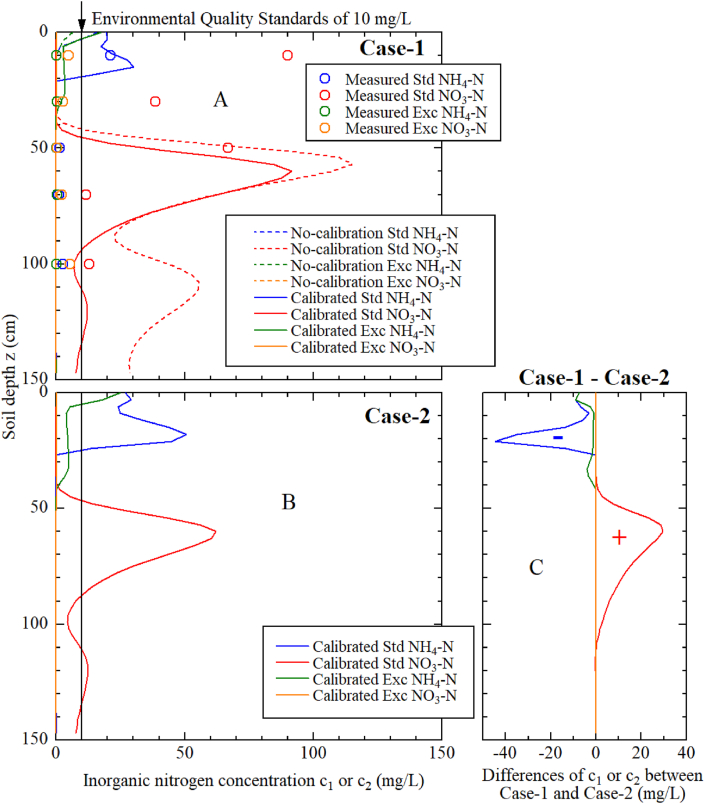
Inorganic nitrogen concentration profiles for Case-1 fertilization conditions (A), inorganic nitrogen concentration profiles for Case-2 (B), and the difference in the concentration distribution between Case-1 and Case-2 (C). Data points, dashed lines, and solid lines represent the measured inorganic nitrogen concentration, calculation results before model calibration, and calculation results using the calibrated model, respectively.

Fig. 7 shows the vertical distribution of the inorganic nitrogen concentration at harvest (t = 157 days) under each fertilization and/or irrigation condition. Regarding the NH_4_–N under Case-1 of the conventional irrigation plot shown in Fig. 7A, the measured and calculated values were consistent. In contrast, the NO_3_–N concentration measured at a depth of 10 cm differed significantly from the calculated results. Excluding this measured value at 10 cm, a vertical distribution with a concentration peak of 70–80 mg L^−^^1^ was observed at depths of 50–60 cm. For NO_3_–N in Case-2 of the conventional irrigation plot (Fig. 7B), a vertical distribution with a concentration peak of 60 mg L^−^^1^ was observed at a depth of 60 cm. Fig. 7C shows the difference in the concentration distribution between Case-1 and Case-2. From these results, the NO_3_–N accumulated around 60 cm could be reduced by 30 mg L^−^^1^ by replacing the fast-acting fertilizer with the slow-release fertilizer.

The measured values of NO_3_–N at a depth of 10 cm differed significantly from the calculated results, likely due to the modeling of the nitrification process (shown in Eq [8]). Although Eq. [8] can accurately reproduce nitrification phenomena after fertilization, it has characteristics that *k*_nit_ asymptotically approaches zero after a long period after fertilization. For this reason, the *k*_nit_ decreased before harvesting (also the soil temperature *T*); however, the nitrification of NH_4_–N remaining in the surface soil could not be reproduced well. Therefore, the improvement of sub-models of the nitrification process is an important topic for future work.

## 4 Discussion

Fig. 6D' and 6E' show the measured NH_4_–N and NO_3_–N concentrations in the excessive irrigation plot reported by Endo et al. (2018c) [26]. The behavior of NH_4_–N at depths of 10–30 cm under excessive irrigation conditions (Fig. 6D and 6D′) was also inconsistent. Specifically, near the soil surface before mid-August, the observed concentration front of NH_4_–N moved to a depth of 30 cm lower than the calculated one, and the absolute value of the observed NH_4_–N concentration was higher than the calculated value. One of the reasons why the NH_4_–N concentration was low in the calculated results may be that the numerical model assumed rapid nitrification. Mineralization and nitrification depend on soil temperature, soil moisture, and elapsed time [35,36]. However, even if the nitrification process is refined, there will inevitably be discrepancies between measured and calculated values due to factors other than nitrification; calibration of the numerical model is necessary for determining the leaching behavior of inorganic nitrogen during the yam growth period.

In contrast, when only H-CDU fertilization shown in Case-2 was applied, a significantly lower concentration distribution was formed compared with the NO_3_–N concentration, as shown in Case-1. In addition, the timing of NO_3_–N leaching shown in Case-2 was similar to that shown in Case-1 since the precipitation and irrigation patterns were similar. Finally, at the harvesting stage (t = 157 days), the NO_3_–N concentration around the depth of 65 cm was lower than 60 mg L^−^^1^. This evidence demonstrated that the NO_3_–N concentration at a depth of 65 cm during the harvesting stage could be halved by setting the fertilizer application system to use the slow-release fertilizer H-CDU rather than using the fast-acting fertilizer. Although numerous studies aimed at controlling the leaching of fertilizer components of slow-release fertilizers that regulate mineralization (Saha et al., 2018) [37] and suppress N_2_O emissions (Amkha et al., 2009) [38], this study is the first to examine the case using numerical analysis.

Under the Case-1 fertilization conditions in excessive irrigation, the NO_3_–N concentration in the soil layer range of 10 < z < 30 cm was highly concentrated immediately before the 70 mm rainfall observed on August 22–23, especially near the depth of 20 cm (NO_3_–N exceeded 200 mg L^−^^1^). After the 70 mm rainfall, NO_3_–N leached to form a concentration distribution of up to 150 mg L^−^^1^ near a depth of 40 cm, and the soil layer range of 30 < z < 50 cm reached a high concentration. Because the apparent depth of the maximum measured NO_3_–N concentration at this time was 70 cm deep, a discrepancy of approximately 30 cm compared with the simulated concentration was recognized. The advection of NO_3_–N contributes greatly to this 30 cm deviation as it is thought that the non-uniformity of soil permeability and water retention, with respect to the depth direction. After the first excessive irrigation under Case-1, NO_3_–N moved further downward by 80 cm, and clear leaching behavior was observed, forming a concentration distribution of up to 50 mg L^−^^1^ near a depth of 120 cm. However, under the fertilization conditions of Case-2, although the leaching behavior of NO_3_–N was similar to that of Case-1, similar to the tendency observed in Case-2 of the conventional irrigation, the maximum NO_3_–N concentration after leaching decreased by approximately half.

From Fig. 5, Fig. 6, it can be observed that the measured value of inorganic nitrogen concentration in soil–water did not perfectly align with the calculated value. Nonetheless, the developed mathematical model was employed to assess the impact of fertilizer selection and irrigation methods on nitrogen leaching in Chinese-yam cultivation. Numerical calculations revealed that extreme and unprecedented torrential rains, with daily precipitation of 200 mm d^−1^, could exacerbate nitrogen leaching and extend its consequences to the surrounding environment. Consequently, it is imperative to establish guidelines for transitioning from the conventional use of fast-release fertilizers to slow-release fertilizers. Furthermore, even when heavy rainfall of less than 200 mm d^−^^1^ occurs, the leaching of nitrate–nitrogen may still occur, depending on the magnitude of advection resulting from precipitation-induced infiltration. It should be noted that this study was conducted in Japan, where the occurrence of daily rainfall of 200 mm d^−^^1^ has been occasionally observed in recent years. Therefore, it is crucial to develop guidelines for the adoption of slow-release fertilizers in order to achieve sustainable agriculture that is in harmony with the environment.

## 5 Conclusion

To enhance the uptake of nutrients by crop roots and maintain a healthy groundwater environment, it is imperative to consider the type and quantity of fertilizer applied, as well as the timing of its application. However, the relationship between the type of fertilizer used for cultivating Chinese yam, the N concentration of soil–water, and the leaching timing remains unknown, particularly under localized torrential rain meteorological conditions. Through an investigation into the behavior of nitrogen components from fast-acting and delayed-release fertilizers in a dune field located in a cold, snowy region, it was discovered that sandy soil with high permeability poses a significant risk for NO_3_–N leaching, particularly during heavy rainfall events. The study reveals that during intense rainfall, NO_3_–N can leach downward up to approximately 80 cm, assuming one heavy rainfall event to be 210 mm. Furthermore, under normal conditions in the absence of localized torrential rains, elevated concentrations of NO_3_–N were observed at depths of 50 < z < 60 cm during Chinese-yam harvesting time, underscoring the influence of weather conditions on nitrogen leaching. Nevertheless, the study demonstrates that the use of slow-release fertilizer (H-CDU) with mineralization during the harvest season can halve the NO_3_–N concentration, showcasing its potential to mitigate nitrogen loss to the environment. These findings underscore the importance of adopting sustainable agricultural practices, particularly in regions prone to extreme weather events. The enhancement and integration of a numerical model facilitate the evaluation of various management strategies, providing valuable insights for farmers and policymakers seeking to minimize environmental impacts while sustaining crop productivity. Ultimately, the study lays a foundation for the development of guidelines that promote sustainable agriculture in regions facing similar environmental challenges.

## Funding

This work was supported by JSPS KAKENHI Grant Number 21H02303 of the Grant-in-Aid for Scientific Research.

## Data availability statement

Data associated with the study have not been deposited into a publicly available repository and will be made available upon reasonable request.

## CRediT authorship contribution statement

**Akira Endo:** Writing – review & editing, Writing – original draft, Visualization, Validation, Supervision, Software, Resources, Project administration, Methodology, Investigation, Funding acquisition, Formal analysis, Data curation, Conceptualization.

## Declaration of competing interest

The authors declare that they have no known competing financial interests or personal relationships that could have appeared to influence the work reported in this paper.
