# Supplementary material for: Dune soil nitrogen leaching for Chinese-yam cultivation: Impact of microbe-decomposable slow-release fertilizer
Source: Heliyon. 2024 May 3;10(9):e30545. doi: 10.1016/j.heliyon.2024.e30545 (PMC11098803; doi:10.1016/j.heliyon.2024.e30545)
Supplement: Multimedia component 1 [file mmc1.docx]

**Supplementary Materials**

Dune soil nitrogen leaching for Chinese-yam cultivation: Impact of microbe-decomposable slow-release fertilizer

Akira Endo*

Faculty of Agriculture and Life Science, Hirosaki University, 3 Bunkyo-Cho,

Hirosaki, Aomori 036-8561, JAPAN

*Corresponding author

Email addresses: aendo777@hirosaki-u.ac.jp (A. Endo)

**The detail of ‘2.4.2. Analytical domain, initial conditions (ICs), and boundary conditions (BCs)’**

The BCs at the soil surface (*z* = 0) are described as follows:

$t>0 J_{w}=P+I \mathrm{at} z=0$ [S1]

$t>0 J_{\mathrm{NH}_{4}-N}=N_{\mathrm{app}}+ J_{w}c_{\mathrm{NH}_{4}-N} \mathrm{at} z=0$ [S2]

$t>0 J_{\mathrm{NO}_{3}-N}={J_{w}c}_{\mathrm{NO}_{3}-N} \mathrm{at} z=0$ [S3]

where *J*_w_ is the water flux density (cm^3^ cm^-2^ d^-1^), *P* is the daily rain (cm d^-1^), *I* is the sprinkler irrigation and/or excessive irrigation (cm d^-1^), $N_{\mathrm{app}}$ is the amount of fertilized nitrogen applied (mg cm^-2^) listed in Table 1 and 2, $J_{\mathrm{NH}_{4}-N}$ and $J_{\mathrm{NO}_{3}-N}$ are the NH_4_-N and NO_3_-N flux densities (mg cm^-2^ d^-1^), respectively, and $C_{\mathrm{NH}_{4}-N}$ and $C_{\mathrm{NO}_{3}-N}$ are the NH_4_-N and NO_3_-N concentrations (mg cm^-3^), respectively, in precipitation.

Regarding the *N*_app_ on the soil surface, the fast-acting fertilizer ammonium nitrogen was applied to the soil surface over 1 day (24 hours). For the H-CDU fertilizer, the mineralization amount per unit time was taken as the nitrogen fertilization amount (**Fig. S4A**), based on its mineralization characteristics at 25 °C. Specifically, after calculating the two parameters obtained by curve fitting using Eq. [S4], Eq. [S5] was obtained by differentiating Eq. [S4] with respect to time and multiplying by the fertilizer application amount. Eq. [S4] is the fitted curve equation using the mineralization characteristics. Then, the obtained value was defined as the mineralized nitrogen amount (as the fertilized amount) on the soil surface per unit time.

$M_{\mathrm{rate}}\left( t \right)=\left\{ {0.84}^{1-m_{2}}-{0.84}^{1-m_{2}}e^{\left[ -K_{2}\left( 1-m_{2} \right)(t-s) \right]} \right\}^{\frac{1}{1-m_{2}}}$ [S4]

$$\frac{dM_{\mathrm{rate}}\left( t \right)}{dt}=M_{\mathrm{rate}}\left( t \right)^{'}=\frac{K_{2}e^{K_{2}\left( m_{2}-1 \right)(t+s)}{0.84}^{1-m_{2}}}{\left[ {0.84}^{1-m_{2}}-e^{K_{2}\left( m_{2}-1 \right)(t+s)}{0.84}^{1-m_{2}} \right]^{\frac{1}{m_{2}+1}+1}} [S5]$$

$N_{\mathrm{mine}}\left( t \right)=N_{H-CDU}M_{\mathrm{rate}}\left( t \right)'$ [S6]

where *M*_rate_ is the mineralization rate of H-CDU shown in **Fig. S4B**, *N*_H-CDU_ is the H-CDU fertilizer amount listed in Tables 1 and 2, *K*_2_ = 0.050 and *m*_2_ = 0.236 are the fitting parameters, *s* is the fertilization day, and *N*_mine_ is the mineralization amount (nitrogen supply amount) of H-CDU per unit time.

The BC for soil temperature is described as follows:

$$t>0, T= T_{0} \mathrm{at} z=0 [S7]$$

where *T*_0_ is the soil temperature at the surface (°C). The daily precipitation *P* (mm d^-1^ converted to cm d^-1^) was applied from the rainfall data observed by Aomori Prefecture. It was assumed that the rainwater at the soil surface would infiltrate downward without loss due to the surface runoff. This soil surface temperature was calculated by considering the soil temperature amplitude and damping depth with respect to the average soil temperature measured at a depth of 10 cm. Specifically, the temperature amplitude at the soil surface was calculated, and the surface temperature was expressed as a function of elapsed time using Eqs. [S8] through [S10].

$$T_{0}\left( t \right)=T_{\mathrm{AVG}}\left( t \right)+A_{0}\sin\left( 2\pi t+\frac{\pi}{2} \right) [S8]$$

$$A_{0}=A_{10\mathrm{cm}}\left\{ \exp\left( \frac{z_{10\mathrm{cm}}}{D} \right) \right\}^{-1} [S9]$$

$$D=\sqrt{{2\kappa}/\omega} [S10]$$

where *T*_AVG_ is the average temperature at *z* = 10 cm (°C), *A*_0_ is the temperature amplitude at the soil surface, *A*_10cm_ is the temperature amplitude at a depth of *z* = 10 cm, *z*_10cm_ is the depth of *z* = 10 cm, *D* is the damping depth (cm), *κ* is the thermal diffusivity, and *ω* is the angular frequency (2*π* / 86400 s).

The BCs at the bottom (z = 150 cm) are described as follows:

$$t>0 J_{w}=K_{u} \mathrm{at} z=150 [S11]$$

$$t>0 -\text{θ}\text{ D}_{\text{1}}\frac{\text{∂}\text{c}_{\text{1}}}{\text{∂}\text{z}}+{J_{w}\text{c}}_{\text{1}}=J_{w}^{150}c_{1}^{150} \mathrm{at} z=150 [S12]$$

$$t>0 -\text{θ}\text{ D}_{\text{2}}\frac{\text{∂}\text{c}_{\text{2}}}{\text{∂}\text{z}}+{J_{w}\text{c}}_{\text{2}}=J_{w}^{150}c_{2}^{150} \mathrm{at} z=150 [S13]$$

$$t>0, \frac{\text{∂T}}{\text{∂z}}= 0 \mathrm{at} z=150 [S14]$$

**Supplementary figures**

**Fig. S1** Nitrification rate coefficient (*k*_nit_) expressed as functions of soil temperature and elapsed time after fertilization (A). Parameter *γ *(*T*) increases exponentially with increasing soil temperature (B).

**Fig. S2** The temporal change in the relative cumulative N-uptake *U*(*t*) and its time derivative Δ*U*(*t*).

**Fig. S3** Initial condition profiles for the volumetric water content (A), inorganic nitrogen (NH_4_-N and NO_3_-N) concentration (B), and soil temperature (C).

**Fig. S4** Mineralization characteristics of H-CDU at 25 °C (A), the mineralization rate *M*_rate_ for each fertilization period (B), and the time derivative of *M*_rate_ (C).

**Fig. S5** Vertical distribution of soil physical properties.


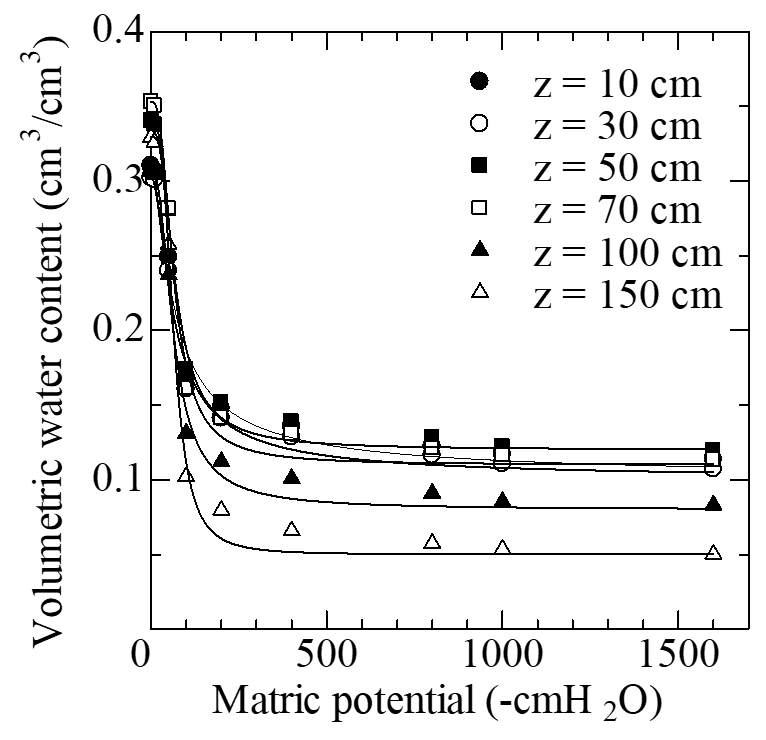


**Fig. S6** Soil water characteristics curve at each depth. The data points and curves represent measured values and fitted curves, respectively.

**Fig. S7** Vertical distribution of soil chemical properties.
